# Five-Year Survival Analysis and Causes of Late Deaths of Infants Admitted to the Tertiary Newborn Intensive Care in Latvia

**DOI:** 10.3390/medicina60020202

**Published:** 2024-01-24

**Authors:** Baiba Balmaka, Sandija Skribāne, Ildze Ābele, Reinis Balmaks

**Affiliations:** 1 Faculty of Residency, Riga Stradinš University, LV-1007 Riga, Latvia; 017855@rsu.edu.lv; 2 Department of Neonatology, Children’s Clinical University Hospital, LV-1004 Riga, Latvia; 3 The Centre for Disease Prevention and Control of Latvia, LV-1005 Riga, Latvia; 4 Department of Anaesthesiology and Intensive Care, Children’s Clinical University Hospital, LV-1004 Riga, Latvia; reinis.balmaks@bkus.lv

**Keywords:** long-term survival, cause of death, epidemiology

## Abstract

*Background and Objectives:* Studies on long-term survival following admission to neonatal intensive care units (NICUs) are scarce. The aim of this study was to analyse the epidemiology, five-year survival, and causes of late death of infants admitted to the only tertiary NICU in Latvia. *Materials and Methods:* The study population included all newborns admitted to the Children’s Clinical University Hospital (CCUH) NICU from 1 January 2013 to 31 December 2017. The unique national identity numbers from the infants or their mothers were used to link the CCUH electronic medical records to the Medical Birth Register and the Database of Causes of Death of Inhabitants of Latvia maintained by The Centre for Disease Prevention and Control of Latvia. *Results*: During the study period, a total of 2022 patients were treated in the tertiary NICU. The average admission rate was 18.9 per 1000 live births per year. One hundred and four patients (5.1%) died in the tertiary NICU before hospital discharge. A total of 131 (6.5%) patients from the study cohort died before 12 months of age and 143 (7.1%) before 5 years of age. Patients with any degree of prematurity had a lower five-year mortality (0.9%, 9 out of 994 discharged alive) than term infants (3.2%, 30 out of 924 discharged alive; *p* < 0.001). Of the 39 patients who died after discharge from the NICU, the most common causes of death were congenital heart disease 35.9% (*n* = 14), multiple congenital malformations and chromosomal abnormalities 17.9% (*n* = 7), cerebral palsy 10.3% (*n* = 4), and viral infections 7.7% (*n* = 3). *Conclusions:* We observed increased mortality up to five years following NICU admission in both premature and term infants. These findings will help to guide the NICU follow-up programme.

## 1. Introduction

Even though neonatal intensive care has been highly effective at improving newborn outcomes, preterm birth remains the leading cause of death in children under five years of age, and neonatal conditions are the leading cause of loss of human capital [1]. Up to 10% of all infants born alive are admitted to neonatal intensive care units (NICU) [2]. The high risk of long-term delayed growth and neurodevelopmental disorders of NICU survivors is well recognized and has led to the establishment of neonatal follow-up programmes [3]. However, less is known about the long-term survival. A recent study from Israel showed that term infants have increased mortality even years after discharge [4]. Overall, studies on the long-term outcomes of neonatal intensive care patients have focused on specific subsets of newborns and lack a population-based perspective. The population-based neonatal (28-day) mortality monitoring programme MBRRACE-UK has allowed identifying regional, social, and ethnic inequalities and thus targeted interventions [5]. The aim of this study was to analyse the epidemiology, five-year survival, and causes of late death of infants admitted to the only tertiary NICU in Latvia.

## 2. Materials and Methods

In Latvia, all critically ill newborns following birth are transferred to one of six perinatal care centres, one of which is linked to a high-risk obstetric unit. However, intensive care following the sixth day of life (i.e., after a perinatal period) and all paediatric subspecialty and surgical care is provided only at the single tertiary paediatric hospital—Children’s Clinical University Hospital (CCUH). CCUH is a stand-alone paediatric hospital not linked to a delivery ward. The study population included all newborns admitted to the CCUH NICU from 1 January 2013 to 31 December 2017. The unique national identity numbers from the infants or their mothers were used to link the CCUH electronic medical records to the Medical Birth Register and the Database of Causes of Death of Inhabitants of Latvia maintained by The Centre for Disease Prevention and Control of Latvia. Prematurity was defined as any case when at least one of the discharge diagnoses was branched from ICD-10 code “P07” (10th revision of the International Statistical Classification of Diseases and Related Health Problems by the World Health Organization). The population denominators were obtained from the Central Statistical Bureau of Latvia’s publicly available annual reports [6]. All analyses were performed using R software version 4.3.0; the survival package was used for Kaplan–Meier analysis [7]. We used a five-year observation period for each patient; therefore, there were no censored cases.

## 3. Results

**Figure 1:**
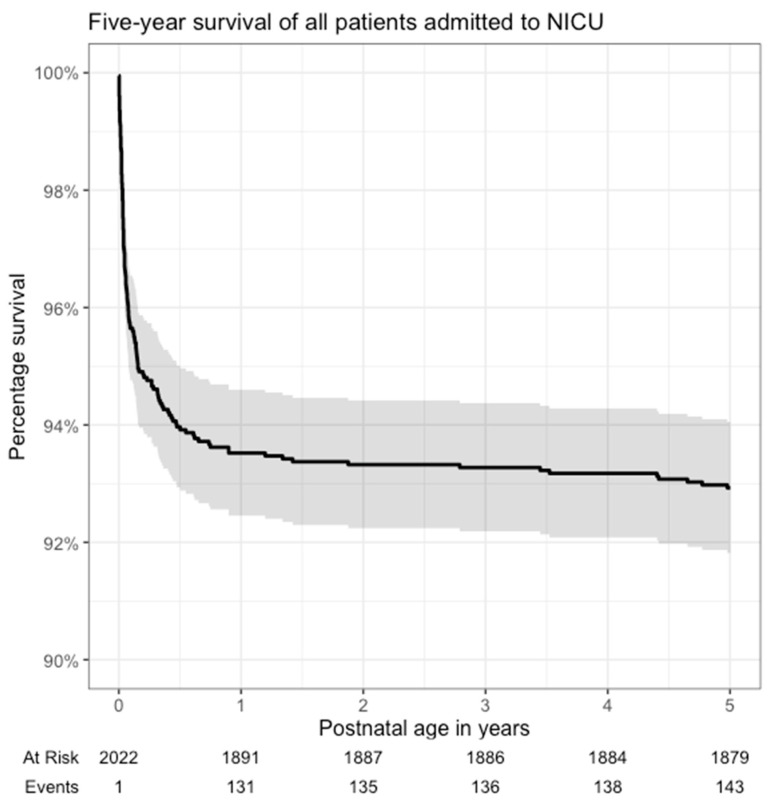
Kaplan–Meier survival analysis. Five-year survival analysis of the entire study cohort. The shaded area denotes a 95% confidence interval.

**Figure 2:**
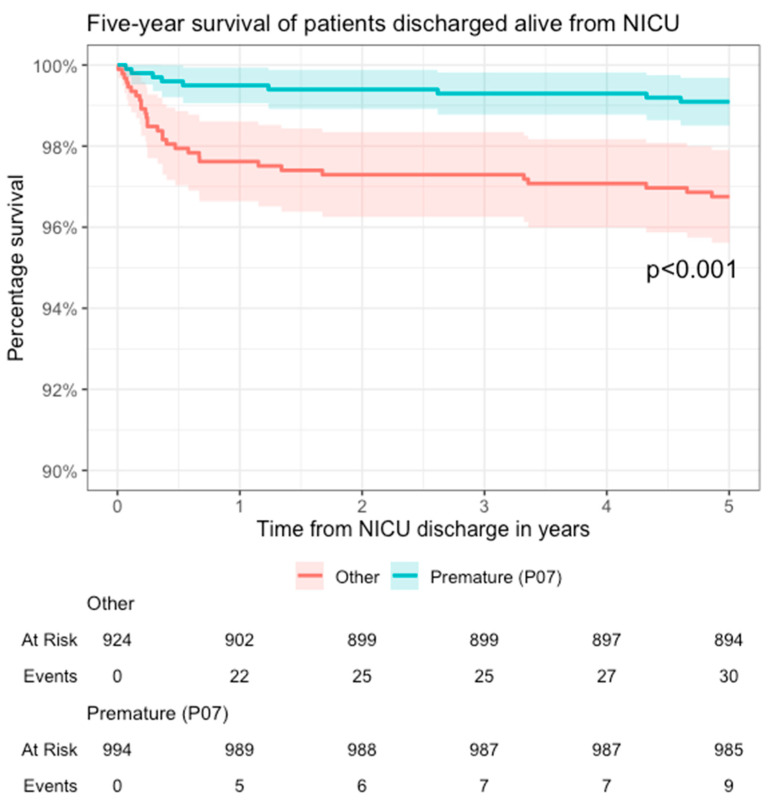
Kaplan–Meier survival analysis. Five-year survival analysis of patients discharged alive from the NICU. The blue line denotes patients with at least one of the discharge diagnoses P07 (ICD-10; “Preterm newborn”); the red line denotes all other patients. The shaded area denotes a 95% confidence interval.

During the study period, a total of 2022 patients were treated in the tertiary NICU. At the same time, 107,117 infants were born alive in Latvia; thus, the average admission rate was 18.9 per 1000 live births per year. One hundred and four patients (5.1%) died in the tertiary NICU before hospital discharge. In the context of the national statistics, 1 of 119 (0.8%) deaths on day of life 0, 25 of 95 (26.3%) from day of life 1 to 6, and 54 out of 74 (73.0%) from day of life 7 to 27 occurred in the tertiary NICU. Of all 288 national neonatal deaths before 28 days of life (2.7 per 1000 live births), 80 (27.8%) were from the study cohort; all before hospital discharge. A total of 131 (6.5%) patients from the study cohort died before 12 months of age and 143 (7.1%) before 5 years of age (shown in Figure 1). Patients with any degree of prematurity had a lower five-year mortality (0.9%, 9 out of 994 discharged alive) than term infants (3.2%, 30 out of 924 discharged alive; *p* < 0.001) (shown in Figure 2). Of the 39 patients who died after discharge from the NICU, the most common causes of death were congenital heart disease 35.9% (*n* = 14), multiple congenital malformations and chromosomal abnormalities 17.9% (*n* = 7), cerebral palsy 10.3% (*n* = 4), and viral infections 7.7% (*n* = 3) (Table 1).

## 4. Discussion

### 4.1. Major Findings

The NICU admission rate (18.9 per 1000 live births) in our study was low compared to the reported 2.75% to 10% in other population-based studies [2,4], which in our case can be explained by the organization of perinatal care in Latvia. The observed NICU mortality was low; however, only a minority of newborns died in the tertiary NICU. This finding requires an assessment of whether patients need to be transferred sooner to the tertiary NICU, where highly specialized treatments such as neonatal surgery and extracorporeal membrane oxygenation (ECMO) are available. The national neonatal mortality in Latvia (2.7 per 1000 live births) was significantly higher compared to the UK (1.65 per 1000 live births) [5]. Similar to the MBRRACE-UK findings, our results warrant exploration into regional and social inequalities, as our group has previously found significant regional variability in paediatric critical illness and mortality in Latvia [8].

The under-five mortality of 7.1% in the NICU population is significantly higher than the population average of 5.4–11 deaths per 10,000 live births during the same period [6]. The 143 deaths in the study population during the observation period approximate to about one-third of all under-five deaths in Latvia during the study years. Furthermore, the survival curve never reached a stable plateau, indicating how vulnerable this population is. Similarly, a study from Israel showed a 20-fold increased risk for death in term infants following NICU admission even up to 15 years following discharge [4]. In Latvia, NICU follow-up is provided for patients for 2 years; however, globally this practice varies from 2 weeks to 3 years [3]. Our findings could suggest a higher end of this range to prevent late deaths. Moreover, the follow-up should target all NICU patients not only premature patients.

With regard to the causes of late deaths, more than one-third were from congenital heart disease. Previously, our group has demonstrated a low five-year survival of patients with univentricular congenital heart disease in Latvia [9], which reflects difficulties in providing high-quality neonatal heart surgery in a country with a small population.

### 4.2. Study Strengths

Due to the low migration effect of the Latvian population, the highly centralized tertiary NICU care, and the unique identification number allocated to each mother and child, we were able to cross-link the medical records and national registries and examine long-term follow-up for mortality.

### 4.3. Study Limitations

There are several significant limitations to the generalizability of these findings. First, the organization of and indications for NICU care may not represent the practice in other countries. Second, we used an administrative dataset with very limited data on obstetric history. Third, the causes of death in some instances were vague (e.g., gastroschisis at age 4). Fourth, we did not analyse other important outcomes such as morbidity, neurodevelopment, and quality of life. Finally, over the last 10 years, several major improvements have been introduced into newborn medicine in Latvia such as the universal adoption of therapeutic hypothermia (2012) and more recently extracorporeal membrane oxygenation (2022). Importantly, the newborn follow-up programme was established in 2015; hence, the data presented in this article are from the pre “follow-up era”, and it is possible that more recent data would demonstrate reduced long-term mortality.

## 5. Conclusions

By linking medical records with national registries, we were able to analyse a population-based epidemiology of five-year survival following admission to the tertiary NICU in Latvia. Thus, this study adds to the little investigated field of the long-term survival of the general NICU population. Furthermore, we provide insights into the causes of late deaths of tertiary NICU patients. In conclusion, we suggest a longer, up to five years, NICU follow-up programme and investigation of mortality in regional perinatal centres where most neonatal, particularly premature, deaths occur.

## Figures and Tables

**Table 1:** Comparison of discharge diagnosis to cause of death for patients discharged alive from the NICU.

Age	Discharge Diagnosis	Cause of Death	*n* = 39
1 month	Congenital heart disease	Congenital heart disease	2
1 month	Infection specific to the perinatal period	Infection specific to the perinatal period	1
1 month	Severe birth asphyxia Bacterial sepsis of newborn	Sudden infant death syndrome	1
1 month	Gastroschisis Congenital heart disease	Congenital heart disease	1
3 months	Congenital heart disease	Congenital heart disease	4
3 months	Other congenital malformations	Trisomy 13	1
3 months	Bacterial sepsis of newborn	Sudden infant death syndrome	1
4 months	Neonatal cerebral ischemia Necrotizing enterocolitis of newborn Bacterial sepsis of newborn	Cerebral palsy	1
4 months	Trisomy 18 Low birth weight newborn, 1500–1999 g	Congenital heart disease	1
5 months	Extreme immaturity of newborn Congenital diaphragmatic hernia	Multiple congenital malformations	1
5 months	Epileptic seizures related to external causes Sepsis of newborn due to Staphylococcus aureus	Disorder of brain	1
5 months	Transient tachypnoea of newborn Other congenital malformations of penis	Multiple congenital malformations	1
5 months	Trisomy 18	Trisomy 18	1
5 months	Congenital heart disease Other bacterial sepsis of newborn	Congenital heart disease	1
6 months	Extremely low birth weight newborn	Metabolic disorder	1
6 months	Multiple congenital malformations Congenital absence, atresia, and stenosis of anus w fistula	Multiple congenital malformations	1
7 months	Congenital heart disease	Congenital heart disease	1
7 months	Preterm newborn Volvulus	Sepsis	1
7 months	Congenital malformation of digestive system Sepsis of newborn due to unspecified staphylococci	Congenital malformation of digestive system	1
8 months	Bacterial sepsis of newborn	Bronchopulmonary dysplasia	1
9 months	Heavy for gestational age newborn	Chromosomal abnormality	1
10 months	Congenital heart disease	Congenital heart disease	1
10 months	Extremely low birth weight newborn, 750–999 g Perinatal intestinal perforation Obstructive hydrocephalus	Other hydrocephalus	1
1 year	Congenital heart disease	Congenital heart disease	2
1 year	Respiratory distress of newborn Low birth weight newborn, 2000–2499 g	Flu	1
1 year	Multiple congenital malformations	Down syndrome	1
2 years	Low birth weight newborn, 1000–1499 g Arteriovenous malformation of cerebral vessels	Viral infection	1
3 years	Acquired periventricular cysts of newborn	Cerebral palsy	1
3 years	Hypoxic ischemic encephalopathy Spastic quadriplegia	Congenital malformation of brain	1
4 years	Gastroschisis Preterm newborn	Gastroschisis	1
4 years	Hypoxic ischemic encephalopathy	Cerebral palsy	2
4 years	Preterm newborn	Flu	1
4 years	Congenital heart disease	Congenital heart disease	1

## Data Availability

The data in this study were obtained from the Children’s Clinical University Hospital and The Centre for Disease Prevention and Control of Latvia where data privacy restrictions apply.
